# Gene signatures derived from transcriptomic-causal networks stratify colorectal cancer patients for effective targeted therapy

**DOI:** 10.1038/s43856-024-00728-z

**Published:** 2025-01-08

**Authors:** Akram Yazdani, Heinz-Josef Lenz, Gianluigi Pillonetto, Raul Mendez-Giraldez, Azam Yazdani, Hanna Sanoff, Reza Hadi, Esmat Samiei, Alan P. Venook, Mark J. Ratain, Naim Rashid, Benjamin G. Vincent, Xueping Qu, Yujia Wen, Michael Kosorok, William F. Symmans, John Paul Y. C. Shen, Michael S. Lee, Scott Kopetz, Andrew B. Nixon, Monica M. Bertagnolli, Charles M. Perou, Federico Innocenti

**Affiliations:** 1 Eshelman School of Pharmacy, University of North Carolina at Chapel Hill, Chapel Hill, NC USA; 2 University of Texas Health Science Center at Houston, Texas, TX USA; 3 USC Norris Comprehensive Cancer Center, Los Angeles, CA USA; 4 Department of Information Engineering, University of Padova, Padova, Italy; 5 Biostatistics and Computational Biology Branch, National Institute of Environmental Health Sciences, Durham, NC USA; 6 Center of Perioperative Genetics and Genomics, Perioperative and Pain Medicine, Brigham & Women’s Hospital, Harvard Medical School, Boston, MA USA; 7 Division of Oncology, University of North Carolina at Chapel Hill, Chapel Hill, NC USA; 8 Lineberger Comprehensive Cancer Center, University of North Carolina at Chapel Hill, Chapel Hill, NC USA; 9 School of Mathematics, University of Science and Technology of Iran, Tehran, Iran; 10 Gamelectronic, Tehran, Iran; 11 University of California at San Francisco, San Francisco, CA USA; 12 Division of the Biological Sciences, University of Chicago, Chicago, IL USA; 13 Department of Biostatistics, Gillings School of Global Public Health, University of North Carolina at Chapel Hill, Chapel Hill, NC US; 14 Department of Microbiology and Immunology, University of North Carolina at Chapel Hill, Chapel Hill, USA; 15 Genentech, South San Francisco, San Francisco, CA USA; 16 Alliance for Clinical Trials in Oncology, Chicago, IL USA; 17 Department of Pathology, University of Texas MD Anderson Cancer Center, Houston, TX USA; 18 Departments of Gastrointestinal Medical Oncology, The University of Texas MD Anderson Cancer Center, Houston, TX USA; 19 Departments of Surgical Oncology, The University of Texas MD Anderson Cancer Center, Houston, TX USA; 20 Duke Center for Cancer Immunotherapy, Duke University, Durham, NC USA; 21 Dana-Farber/ Partners Cancer Care, Harvard Medical School, Boston, MA USA; 22 Department of Genetics, School of Medicine, University of North Carolina at Chapel Hill, Chapel Hill, NC USA

**Keywords:** Colorectal cancer, Computational biology and bioinformatics, Genetic interaction, Tumour biomarkers, Cancer genomics

## Abstract

**Background:**

Gene signatures derived from transcriptomic-causal networks offer potential for tailoring clinical care in cancer treatment by identifying predictive and prognostic biomarkers. This study aimed to uncover such signatures in metastatic colorectal cancer (CRC) patients to aid treatment decisions.

**Methods:**

We constructed transcriptomic-causal networks and integrated gene interconnectivity into overall survival (OS) analysis to control for confounding genes. This integrative approach involved germline genotype and tumor RNA-seq data from 1165 metastatic CRC patients. The patients were enrolled in a randomized clinical trial receiving either cetuximab or bevacizumab in combination with chemotherapy. An external cohort of paired CRC normal and tumor samples, along with protein-protein interaction databases, was used for replication.

**Results:**

We identify promising predictive and prognostic gene signatures from pre-treatment gene expression profiles. Our study discerns sets of genes, each forming a signature that collectively contribute to define patient subgroups with different prognosis and response to the therapies. Using an external cohort, we show that the genes influencing OS within the signatures, such as *FANCI* and *PRC1*, are upregulated in CRC tumor vs. normal tissue. These signatures are highly associated with immune features, including macrophages, cytotoxicity, and wound healing. Furthermore, the corresponding proteins encoded by the genes within the signatures interact with each other and are functionally related.

**Conclusions:**

This study underscores the utility of gene signatures derived from transcriptomic-causal networks in patient stratification for effective therapies. The interpretability of the findings, supported by replication, highlights the potential of these signatures to identify patients likely to benefit from cetuximab or bevacizumab.

## Plain language summary

Response to cancer treatment varies greatly among patients. To improve outcomes, it is crucial to identify patients who are more likely to respond well to particular treatments. Changes in gene expression within cancer cells lead to alterations in cellular behavior, which can influence the response to treatment. In this study, we investigated how these changes affect patient outcomes. We identified specific gene expression patterns in patients who benefited from certain treatments. These findings could help guide treatment decisions, enabling personalized therapies that are more likely to be effective and improve patient outcomes.

## Abstract (web summary)

Yazdani et al. identify gene signatures by integrating transcriptomic-causal networks into overall survival analysis, providing interpretable biomarkers for patient stratification. Gene signatures are found in metastatic colorectal cancer that indicate patients with specific profiles might benefit from cetuximab or bevacizumab.

## Introduction

Targeted therapies have emerged as a promising therapeutic option in the management of metastatic colorectal cancer (mCRC) when used in combination with chemotherapy^1,2^. However, the efficacy of targeted therapies can vary significantly among individuals due to their biological heterogeneity^3^. Within the complex landscape of cancer biology, multiple genes and their interconnected pathways collaborate to influence tumor behavior and the response to therapy. Integrating gene interactions into gene expression profiling yields insights into cancer biology^4–8^ and their connection to clinical outcomes such as OS can indicate their potential for direct clinical utility in guiding clinical care and treatment^9^.

Here, we identify gene interconnectivity by constructing a transcriptomic-causal network, which is a Bayesian network augmented with the principles of Mendelian randomization^10–13^. By integrating gene interconnectivity into the OS analysis, we estimate the effect of gene expression on OS while controlling for confounding genes. Additionally, the constructed causal network facilitates a data-driven approach for the discovery of a group of genes (subnetwork) that can form a gene signature for patient stratification. The small set of functionally related genes within a signature enhances the interpretability and reproducibility of the results, contributing to more targeted and effective therapeutic strategies in managing colorectal cancer.

We identify promising predictive and prognostic gene signatures using data from a randomized phase III trial (CALGB/SWOG 80405)^14^ comprising 1165 patients with mCRC treated with cetuximab or bevacizumab combined with chemotherapy (FOLFOX or FOLFIRI) as first-line therapies. This large clinical trial provides a unique opportunity to investigate pre-treatment gene expression profiles in each arm of the study and stratify patients who benefit more from their assigned treatments. We replicate the findings using data from 103 patients initially excluded from the main analysis along with external cohorts, including the Cancer Genome Atlas (TCGA). Using an external protein-protein interaction database^15^, we assess the biological relevance of interconnectivity among genes. Furthermore, we link these signatures to Consensus Molecular Subtypes (CMS) of CRC^16^ and immune features as defined in the literature, which serve as key inputs to a description of the immune subtypes of cancer.

This study underscores the utility of gene signatures derived from transcriptomic-causal networks in stratifying patients for effective targeted therapy. Additionally, it offers promising predictive biomarkers to identify individuals who are more likely to respond positively to cetuximab or bevacizumab, as well as those who may potentially exhibit resistance to these therapies. These findings contribute toward tailoring treatment strategies for personalized medicine.

## Methods

This study is conducted on a subset of CRC patients of European descent enrolled in a randomized phase III trial CALGB/SWOG 80405 due to the limited sample size from other ethnicities. Germline genotyped data were extracted from the peripheral blood of 1165 patients (Fig. S1). RNA-seq data were extracted from the primary tumor tissue of 469 patients, obtained from pre-treatment formalin-fixed paraffin-embedded blocks (Fig. S2). The trial was designed to compare cetuximab, bevacizumab, or cetuximab + bevacizumab, each plus chemotherapy, as first-line therapy in KRAS wild-type advanced or mCRC^17^. This study investigates pre-treatment gene expression profiles in the bevacizumab and cetuximab arms of the study and identifies predictive gene signatures to subgroup patients who benefit more from their assigned treatments.

To enhance the reproducibility and interpretability of the findings, we integrate gene interconnectivity into the OS analysis and account for the potential confounding effects of genes. We achieve this by constructing transcriptomic-causal networks, which are Bayesian networks augmented with genetic information based on Mendelian randomization principles^10,12^. We further conduct expression quantitative trait loci (eQTL) analysis and utilize the eQTLs as potential instrumental variables to construct the networks and estimate causal relationships among genes. This analysis is performed using data from patients treated with either bevacizumab or cetuximab. We assess the stability of the network and replicate the edges using 103 samples from the bevacizumab + cetuximab arm of the study (Methods). Furthermore, we replicate the interconnectivity among genes using protein-protein interaction data from STRING^15^.

**Fig. 1:**
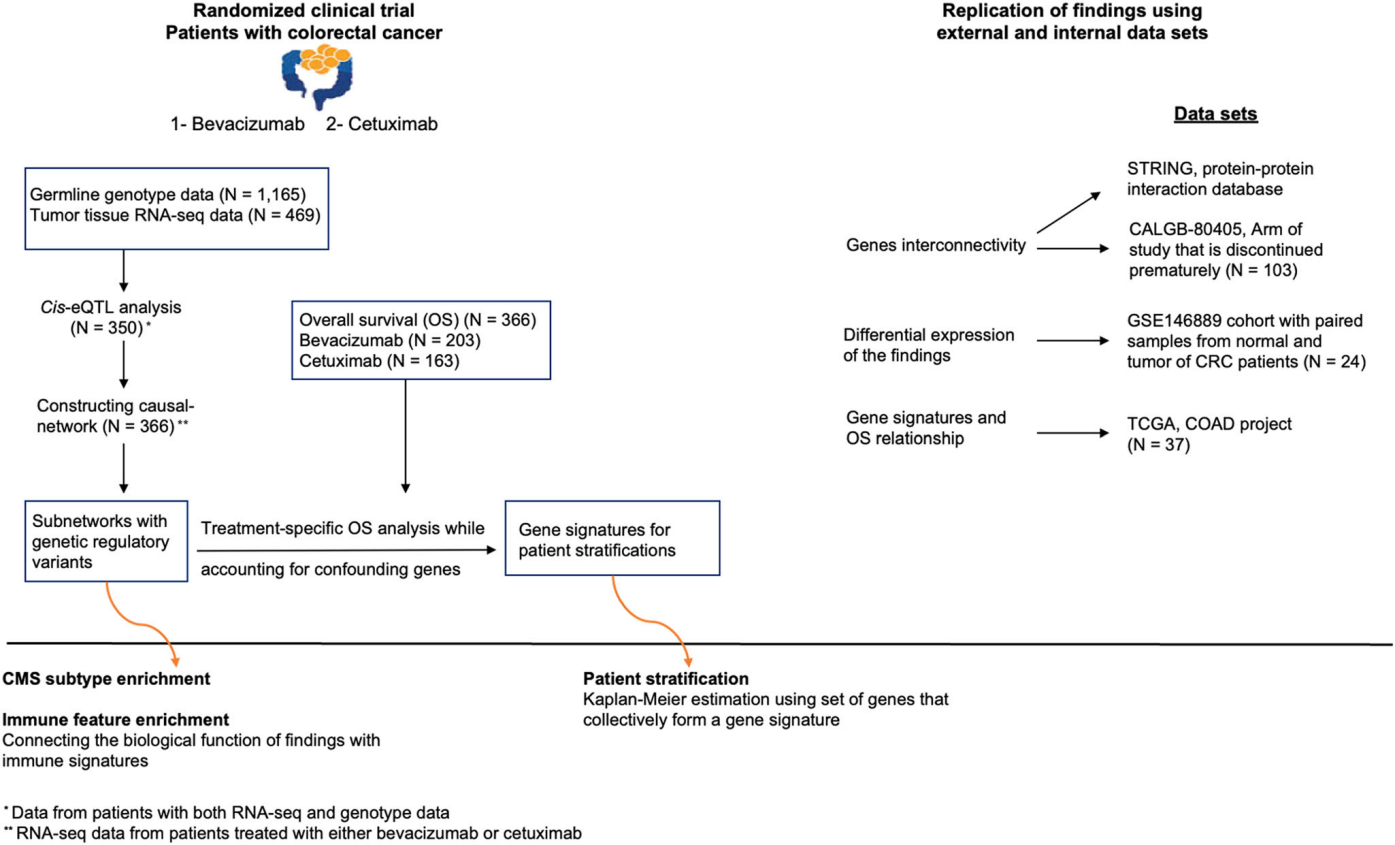
Overall workflow. The uncovered gene interconnectivity through the construction of a transcriptomic-causal network was integrated into the OS analysis to address the confounding effects of genes. This integration allowed us to identify gene signatures that collectively impact OS. These gene signatures were used for patient stratification based on their OS. The results were replicated across multiple replication sets. To elucidate the biological functions of the findings, we explored the association of the identified genes with immune signatures and CMS subtype of CRC. We also investigated the functional relationships among genes based on the protein-protein interactions database.

We identify subnetworks and define a signature as a set of genes associated with OS within a subnetwork. We replicate the findings using two external cohorts in addition to a subset of data from the cohort under study that was not used in previous analyses. Next, we relate the signatures to the CMS subtypes of CRC to enhance their clinical relevance. Additionally, we explore the relationship between our findings and immune signatures defined in the literature as key inputs to describing the immune subtypes of cancer. Figure 1 represents the overall workflow of the study.

For the analyses reviewed here, we account for the influence of the tumor microenvironment by adjusting for immune cell abundances estimated from RNA-seq data. In addition, all the analyses are adjusted for all RAS and BRAFv600E mutation status along with age, gender. The eQTL analysis is adjusted for batch effects.

### Data

Patients in this study were drawn from the Cancer and Leukemia Group B (CALGB; now a part of the Alliance for Clinical Trials in Oncology) and SWOG 80405 (Alliance) trial. The trial was initiated in September 2005 with a total of 2326 patients randomized to the three treatment arms (bevacizumab, cetuximab, or their combination in addition to chemotherapy with FOLFIRI or FOLFOX). While institutional review board approval was required at all participating centers and all participating patients provided written informed consent^17^, this study has used deidentified samples.

#### Genotyping

DNA was extracted from peripheral blood of 2334 patients. The first genotyping batch was performed on the Illumina HumanOmniExpress-12v1 platform at the Riken Institute (Tokyo, Japan) and included 731,412 genotyped variants. The second genotyping batch was performed on the Illumina HumanOmniExpress-8v1 and included 964,193 SNPs. A total of 719,461 SNPs from HapMap from batch 1 were also on the chip from batch 2. The quality control was performed to remove SNPs with mismatched annotation between the two platforms, genotyping call rates less than 99%, departures from Hardy–Weinberg equilibrium (*P* < 10^−8^), allele frequencies less than 0.05, and individuals with genotyping call rate <0.90. A total of 540,021 SNPs genotyped for 1165 samples remained^18^ after passing these filters (Fig. S1).

#### Tumor RNA sequencing

Tumor RNA was extracted from pre-treatment formalin-fixed paraffin-embedded (FFPE) tumor blocks (96% primary, 2% metastatic, 2% unknown) from 584 CALGB/SWOG 80405 patients. TruSeq RNA access target enrichment and library preparation protocol were performed using 250 ng of template RNA. Sequencing was done using synthesis chemistry targeting 50 M reads with a read length of 2 × 100 bp per sample on the HiSeq 2500. Data processing was conducted using standard procedures described by Kalari et al.^19^.

#### Clinical outcomes and covariates

The primary endpoint of OS was calculated from the time of study entry to death or the last known follow-up for those without reported death. The median follow-up of 640 samples in bevacizumab and cetuximab arms was 65.7 months (95% confidence interval, 63.5–70)^17^. In addition, *BRAF* and all *RAS* mutation status were determined by BEAMing (beads, emulsion, amplification, magnetics; Hamburg, Germany) technology^20^ and included in the analysis as covariates in addition to age and gender.

### Data preprocessing

Among the 584 samples with RNA-seq data, the majority (86%) were European American, with 9% being African American and 5% from other ethnicities. Due to the small sample size of the other ethnicities, our analysis focused on primary tumor samples from European American. To ensure data quality and reliability, we excluded genes with low expression variation (standard deviation <0.5) and low counts across the samples (>30% zeros). This resulted in a final set of 8301 genes for further analysis. we applied upper quartile normalization, which enabled comparability of gene expression values across different samples. We removed duplicated samples (*n* = 5) and tumors with low gene expression across the genome (>50% genes with zero counts; *n* = 1). Further details and visuals can be found in Figs. S2, S3. We then transformed the RNA-seq data into the log2 scale for the analysis.

To assess the presence of batch effects or hidden population stratification in the RNA-seq data, we conducted principal component analysis (PCA) (Fig. S4). In order to validate the self-reported gender information, we utilized k-means clustering based on the expression patterns of genes on chromosome Y. This analysis revealed that 5 samples had a discrepancy between their recorded gender and their biological sex (Fig. S5).

Given the influence of the tumor microenvironment on tumor biology, it is crucial to consider the heterogeneity of cell types within the tumor samples when analyzing RNA-seq data. The tumor microenvironment consists of various cell types, including tumor cells, immune cells, stromal cells, and others, which can have distinct gene expression profiles. Therefore, correcting for the abundance of different cell types becomes even more important in order to accurately capture the gene expression patterns specific to tumor cells and to account for any confounding effects introduced by non-tumor cell types. To this end, we estimated the abundance of immune cell types in our RNA-seq data using CIBERSORTx^21^ with the validated leukocyte gene signature matrix as a reference. We defined a cell phenotype to be enriched in our data if at most 30% of its estimated scores across samples are zero and its standard deviation is greater than 0.1. As a result, 9 hematopoietic cell phenotypes were enriched in our data: naive and memory B cells, plasma cells, CD8+T cells, resting and activated memory CD4+T cells, M0 and M2 macrophages, and activated mast cells^22^. The relationships between the immune cell types and immune signatures are represented in Supplementary Fig. S6.

We used 1165 samples with 540,021 SNPs for imputation and employed phased haplotypes from the Haplotype Reference Consortium (HRC) panel through the University of Michigan Imputation Server^23^ (https://github.com/statgen/locuszoom-standalone/). Phasing was done using Eagle v2.4 algorithm^24^. The HRC panel combines sequence data across >32,000 individuals from >20 medical sequencing studies. The imputed genotype data with imputation score >0.7 and minor allele frequency (MAF) > 0.05 included 5,539,144 common SNPs. These SNPs were used in all the downstream analyses.

For analysis that includes pre-treatment or baseline data (samples with genotype and RNA-seq), we used samples in all arms. However, for the analysis that involved post-treatment data (samples with overall survival and events), we excluded Arm 3 that showed shorter overall survival with two other arms^17^ (Fig. S7). The comparisons between the population with the RNA-seq and the population without it are presented in Supplementary Data 1.

### *cis*-eQTL analysis

To identify germline genetic variants associated with tumor gene expression, we focused on *cis*-eQTL since gene expression is affected by nearby genetic variations^12,25^. Therefore, for all pairs of genes and SNPs within 1 Mb upstream and downstream of the genes’ transcription start sites (TSS), we applied a linear regression model. In our primary analysis, we estimated latent variables for the potential confounders using the probabilistic estimation of expression residuals (PEER) approach^26^. However, PEER factors did not explain the variation in RNA-seq data in our study (more details in Supplementary Method and Figs. S8–10).

To address the impact of heterogeneous cell types in our RNA-seq data and to mitigate potential batch effects, we applied several adjustments. We adjusted the expression level of the genes for the enriched cell types in our data estimated by CIBERSORTx^21^. Additionally, we incorporated the first principal component of genotype data to remove the contribution of batch effects that may have arisen during sample processing and sequencing (Fig. S11). Furthermore, we considered important covariates such as gender, age, and mutation status (including RAS and BRAFv600E) in our analysis to account for potential confounding factors. This analysis was performed using FastQTL^27^. We applied the adaptive permutation mode of FastQTL while setting for 10,000 permutations and selected eGenes with at least one *cis*-eQTL with an adjusted *p* value < 0.05 at the gene level. These genes are the ones selected for the OS analysis.

### Enrichment analysis in genomic regions

We investigated the enrichment of identified *cis*-eQTLs in the biological location in DNA, including genic, intron, exon, intergenic, distal intergenic, and upstream and downstream (≤300 bp) of a gene. To demonstrate that the number of *cis*-eQTLs in any region is higher than expected by chance, we simulated the null distribution by permuting 1000 random sets of SNPs with the size of *cis*-eQTL matching *cis*-eQTL in terms of allele frequency, gene density, distance from TSS, and density of tagSNPs arising from genomic variability of linkage disequilibrium^28^. We then calculated the Z-score for the observed number of *cis*-eQTL in each region based on the simulated null distribution.

We also looked for the overlap of *cis*-eQTLs with enhancer database from the Roadmap Epigenomics Consortium^29^. In particular, we focused on active, genic, and bivalent chromatin states in colon sigmoid, mucosa, and smooth muscle. An active enhancer refers to the regulatory region of DNA that interacts with the promoter DNA region; a genic enhancer refers to regulatory regions in a gene; a bivalent enhancer refers to segments of DNA that have both repressing and activating epigenetic regulators in the same region. We counted the number of top *cis*-eQTLs (the most significant associated SNP per gene) that lie within enhancer sequences in each tissue. We calculated the z-score for each tissue similar to the previous section (Enrichment in the genomic region) and tested the significant levels.

### Transcriptomic-causal networks

The transcriptomic-causal networks are data-driven networks augmented with the principles of Mendelian randomization (MR). The use of transcriptomic-causal networks enables us to uncover the intricate biological connections between genes and identify confounding genes in order to evaluate the direct impact of a gene on overall survival (OS). For the feasibility of constructing robust networks, we initially employed *k*-mean clustering and clustered genes in 4 classes. We then built a data-driven network for each cluster based on an order-independent implementation of the conditional independence properties, i.e., directed acyclic graph (DAG), learning PC-algorithm^10^. We also augmented the networks with eQTLs as instrumental variables (IVs) to identify causal networks established in the MR principles^12,30–32^. We identified stable networks by employing two different techniques and then focused on the edges commonly identified by both methods. One method was based on false discovery rate (FDR) where we built a dense network by retaining all the edges within each cluster. We then select significant edges between gene pairs with FDR ≤ 0.05.

The other method for the network stability determination was based on the Hamming distance metric (HD). In this method, we constructed the networks for different values of ${\alpha }_{i}\in \left\{{10}^{-i}{|\; i}=3,\ldots ,22\right\}$, where ${\alpha }_{i}$ represents the significance level used in statistical tests to determine conditional dependencies between two genes, given the expression levels of other genes in the network. We then calculated HD for each pair of networks ($H{D}_{{ij}}$) where $j=i-1$. Smaller ${\alpha }_{i}$ leads to a sparser network, but the question is which ${\alpha }_{i}$ yields the network corresponding to the actual sparsity level. To answer this question, we employed a piecewise regression model by regressing $H{D}_{{ij}}$ on ${\alpha }_{i}$ as follows:

1 $$H{D}_{{ij}}={\beta }_{0}+{\beta }_{1}{\alpha }_{i}+{\beta }_{3}\left({\alpha }_{i}-{\alpha }_{k}\right){I}_{\left\{{\alpha }_{k} < {\alpha }_{i}\right\}}$$

Here, ${I}_{\{{\alpha }_{k} < {\alpha }_{i}\}}$ is the indicator function of significant slope change for the breakpoint ${\alpha }_{k}$. The breakpoint represents a point that indicates a significant change in the slope of the regression model. By fitting this model for all possible breakpoints, *α*_*k*_s, we identified the optimal ${\alpha }_{i}$ corresponds to the maximum breakpoint. Fig. S12 represents this procedure for all the clusters in the analysis.

#### Validating the edges in the network

To validate the interconnectivity among genes, we used 103 additional patients for the combination arm of the study (bevacizumab + cetuximab plus chemotherapy) excluded from the main analysis. We considered this set as the test set and replicated the interconnectivity among genes using the predictive linear model as follows:

$${{\hat{g}}_{{test}}={G}_{{test}}({G}^{{\prime} }G)}^{-1}{G}^{{\prime} }g$$

Here, $G$ is an $n\times p$ matrix of the expression level of $p$ predictors for *n* samples used for building the network. The predictors of each gene ($g$) refer to its direct upstream and downstream genes in the network. ${G}_{{test}}$ is an *m* × *p* matrix of predictors’ expression levels for ${m}$ samples (here, *m* = 103) selected for the test set. We then calculated the correlation between observed $({g}_{{test}})$ and predicted (${\hat{g}}_{{test}}$) values, and considered a link validated if the correlation was above 0.5.

#### Identification of sub-networks and their regulatory genes

We defined a sub-network as a set of genes directly connected to an eGene or after one step. We used the eQTLs to implement the Mendelian randomization (MR) technique in addition to the rule of v-structure^12,30,31^ (Fig. S13) for identification of causal relationship between genes. The causal relationships discovered genes with a high potential of having a regulatory role in the sub-networks. In addition, we included known gene regulatory pathways for identifying the causal network. For instance, it is known that *FANCI* and *BLM* are in the Fanconi anemia pathway, and *FANCI* regulates *BLM*^33^. Therefore, we used this knowledge to define causal relationship between these two genes in sub-network 3. There were some edges with unidentified directions, but we did not remove those from the study.

#### Identifying gene signatures impacting OS

We performed the Cox proportional hazard model for genes in each sub-network, considering the underlying relationship of the genes in the network by adjusting the analysis for the impact of confounding genes on the gene-OS relationship as

$${h}_{l}\left(t\right)={h}_{0}(t)\exp [\alpha +{{\boldsymbol{\gamma }}}\widetilde{G}+{{\boldsymbol{\beta }}}G+{{\boldsymbol{\theta }}}Z+{{\boldsymbol{\delta }}}T]$$

where $\widetilde{G}=({\widetilde{g}}_{1},\ldots ,\,{\widetilde{g}}_{q})$, ${\widetilde{g}}_{i}={g}_{i}\,(I-{G}_{i}{({G}_{i}^{T}{G}_{i})}^{T}{G}_{i}^{T})\,,\,G=({G}_{1},\,\ldots ,\,{G}_{q})$. Here, ${G}_{i}$ includes all the upstream genes of ${g}_{i}$ in the sub-network, Z represents a matrix of covariates, and T represents a matrix of enriched cell types. This analysis estimated the effect of ${g}_{i}$ on OS since it included their adjusted expression level, ${\widetilde{g}}_{i}$, for the effect of their upstream genes in the sub-network.

In each sub-network, we defined a gene signature as the set of genes with significant impacts on OS, considering that these genes collectively influence OS outcomes. To streamline the process of nominating a scoring model for prospective testing as a gene signature and to aid in visualizing survival plots, each patient was categorized as either ‘high’ or ‘medium-to-low’ for a particular gene. This categorization depended on whether the patient’s gene expression value exceeded the third quartile of gene expression or fell below it, respectively.

Determining the third quartile of gene expression involved considering the gene expression values across the entire patient cohort, irrespective of the treatment received by individual patients. This approach enhances the generalizability of patient stratification. For a given patient, a gene signature was labeled as “high” if all its constituent genes were categorized as “high” based on the aforementioned criterion. Conversely, it was labeled as “medium-to-low” if all genes within the signature were categorized as “medium-to-low”.

Subsequently, we estimated the survival function using the Kaplan–Meier estimator to analyze the survival outcomes associated with these gene signatures.

### Reporting summary

Further information on research design is available in the Nature Portfolio Reporting Summary linked to this article.

## Results

### Genetic regulatory variants of gene expression in CRC tumor tissue

To identify candidate susceptibility genes subject to regulation by genetic variants, we performed a *cis*-eQTL analysis on 8301 genes with adequate variation (standard deviation of normalized counts across samples >0.5) while adjusting for covariates. The relationship of 33,209,829 *cis*-eQTL-gene pairs was assessed using 350 samples with both RNA-seq and genotype data. After applying the permutation test (*p* value < 0.05), we selected the top 352 genes (Figs. S14, S15, and Supplementary Data 2). The enrichment analyses of eQTL annotation and enhancers are presented in supplementary material (Fig. S16).

### Transcriptomic-causal network

We combined the samples of bevacizumab and cetuximab arms to identify the transcriptomic-causal network since RNA seq data were recorded prior to the treatments (Fig. S17). We identified the network on 8301 genes and focused on 2267 edges that passed the stability assessment. Using the 103 patients (treated with bevacizumab + cetuximab), who were initially excluded from the main analysis, we successfully replicated 71% of the interconnectivity among genes (“Methods” and Figs. S18–20). By taking these steps, we reduced the likelihood of false positive discoveries and increased the chances of reproducibility for the identified edges. We then integrated *cis*-eQTL analysis results for sub-networks identifications and the application of Mendelian randomization technique to identify causal relationships among genes (“Methods”).

### Gene signatures and patient stratification

**Fig. 2:**
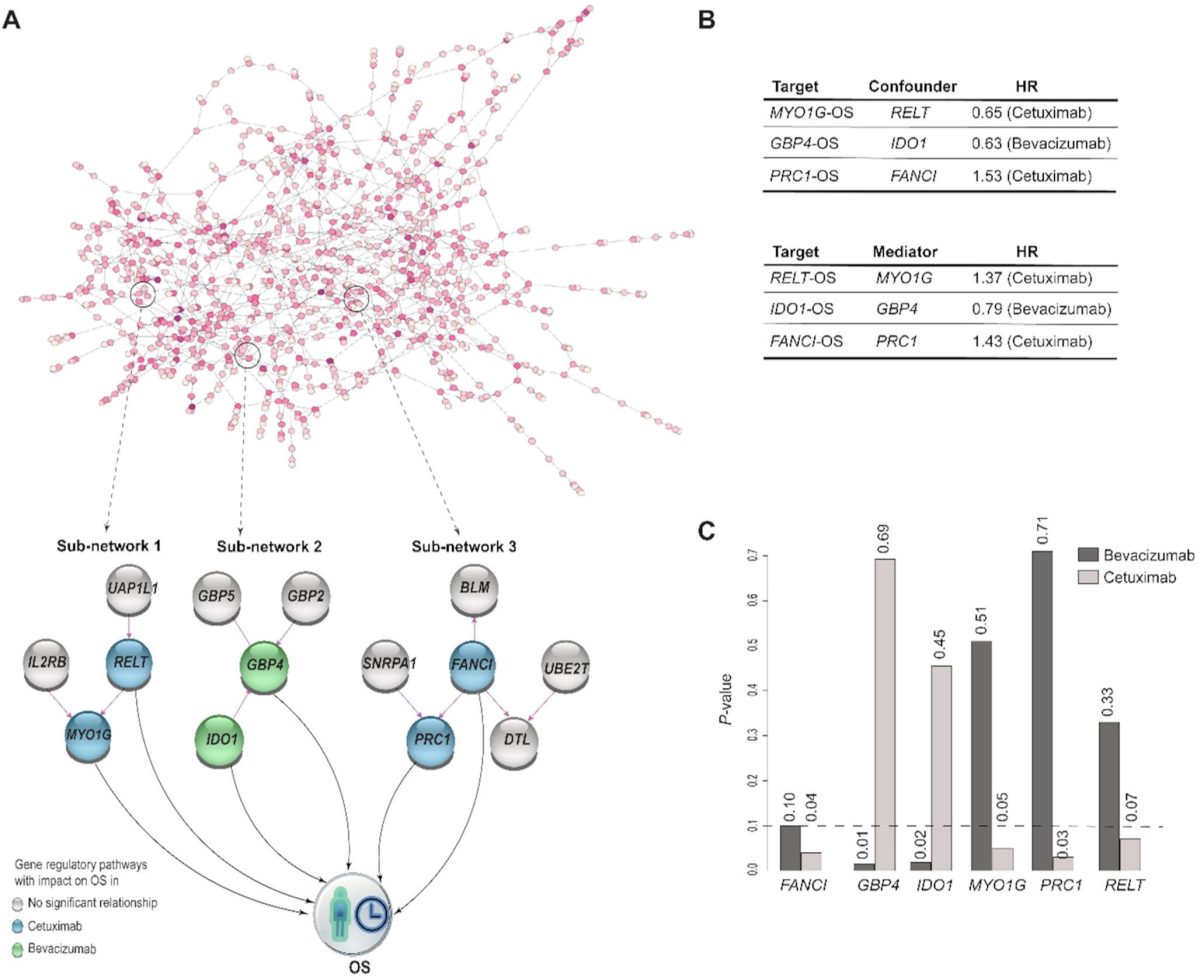
Transcriptomic-causal network linked to OS. **A** Revealed transcriptomic-causal network and its sub-networks comprising genes with significant effects on OS. **B** Confounder genes and mediators respect to OS. **C** Treatment specific *p* values for genes with influencing OS within the sub-networks, with significant level represented with dashed line.

We investigated the effect of the gene expression levels on OS by integrating the transcriptomic-causal network with Cox-proportional hazard models for the patients receiving bevacizumab (203 samples) and cetuximab (163 samples) separately. In this way, we controlled for the effects of confounding genes identified using the network. This analysis yielded the identification of three gene signatures corresponding to three sub-networks that comprised genes with significant effects (*p* value < 0.1) on overall survival (OS) for either bevacizumab or cetuximab treatments (Fig. 2).

One of the sub-networks (Fig. 2A, sub-network 1) includes 4 genes (*UAP1L1, IL2RB, RELT*, and *MYO1G*). Among these genes, *MYO1G* was identified as an eGene. To assess the effect of *MYO1G* on OS, we controlled for the effect of *RELT* as confounding gene and observed that *MYO1G* prolonged, and *RELT* shortened *OS* under cetuximab treatment (HR: 0.65, 1.37, *p* value: 0.05, 0.07, respectively) but not under bevacizumab treatment (HR: 0.86, 1.14, *p* value: 0.51, 0.33, respectively), (Fig. 2B, C).

The other sub-network (Fig. 2A, sub-network 2) involved 4 genes (*IDO1*, *GBP2*, *GBP4*, and *GBP5*), three of which belong to the guanylate-binding protein (GBP) family, including eGene *GBP5*. We observed that the two directly connected genes *IDO1* and *GBP4* significantly prolonged OS of patients under bevacizumab treatment (HR: 0.79, 0.63, *p* value: 0.018, 0.015, respectively) and not under cetuximab (HR: 0.92, 0.93, *p* value: 0.455, 0.692, respectively), (Fig. 2B, C). In this analysis, we controlled for the confounding role of *IDO1* on the *GBP4*-OS relationship.

The third sub-network (Fig. 2A, sub-network 3) includes 6 genes (*BLM, FANCI, UBE2T, SNRPA1, PRC1*, and *DTL*), with *BLM* being an eGene. We observed that *PRC1* and *FANCI* shortened the OS of patients treated with cetuximab (HR: 1.53, 1.43; *p* value: 0.03, 0.04, respectively) but not with bevacizumab (HR: 0.94, 1.31; *p* value: 0.71, 0.10), (Fig. 2B, C).

**Fig. 3:**
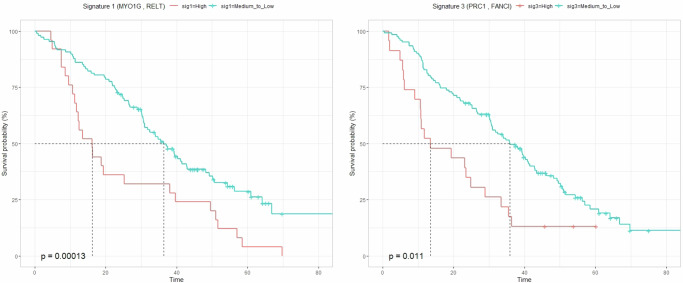
Survival curves stratifying patients by identified gene signatures. Kaplan-Meier plots for signatures 1 and 3, with “high” indicating all the genes within the signatures are with high expression levels, “medium-to-low” indicating all the genes within the signatures are with medium-to-low expression levels. The turquoise curve represents OS for patients with a medium-to-low signature level, while the red curve represents those with a high signature level. The median OS decreased notably from 36.4 to 16.1 months (*p* value: 0.0001) for signature 1 and from 35.8 to 13.5 months (*p* value: 0.011) for signature 3, showing a significant difference between medium-to-low and high expression levels.

To facilitate the nomination of a scoring model for prospective testing as a gene signature and to support the visualization of survival plots, we dichotomized each gene into “high” and “medium-to-low” expression levels. The cutoff was defined based on the third quartile of expression of a gene across all the patients in this cohort regardless of the given treatment. For a given patient, a signature was labeled as “high” if all its genes were classified as “high” expression after dichotomization, and as “medium-to-low” if all the genes within the signature were classified as “medium-to-low” expression. We then estimated the survival function using the Kaplan–Meier estimator (“Methods”). We observed a significant decrease in the median OS from 36.4 to 16.1 months (*p* value: 0.0001) for patients with high vs. medium-to-low expression levels for signature 1. This signature comprises genes *RELT* and *MYO1G* from sub-network 1. Similarly, for signature 3, which comprises genes *FANCI* and *PRC1* from sub-network 3, we observed a significant reduction in median OS from 35.8 to 13.5 months (*p* value: 0.011) for patients with high versus medium-to-low expression levels for signature 3, as illustrated in Fig. 3. For patients with high expression for one gene and medium-to-low for another gene within the signatures see Fig. S21. Due to the limitations associated with dichotomizing the data in Kaplan–Meier estimators, the statistical power of the analysis for sub-network 2 in the bevacizumab arm was insufficient to detect significant differences among patients.

To investigate the potential role of the identified gene signatures in treatment efficacy and resistance, we linked these signatures to CMS subtypes in colorectal cancer. This analysis revealed that 77% of patients with the CMS3 subtype treated with bevacizumab exhibited medium to low levels of both *GBP4* and *IDO1*. These patients, with a median OS of 14.4 months (95% CI: 10.9–17.8), are more likely to show resistance to the bevacizumab treatment regimen. Similarly, the majority of patients with the CMS2 subtype (79%) showed medium to low expression levels for both *FANCI* and *PRC1*, indicating a higher likelihood of benefiting from cetuximab (median OS: 39.2; 95% CI: 34.9–45.6).

### Immune feature enrichment

**Fig. 4:**
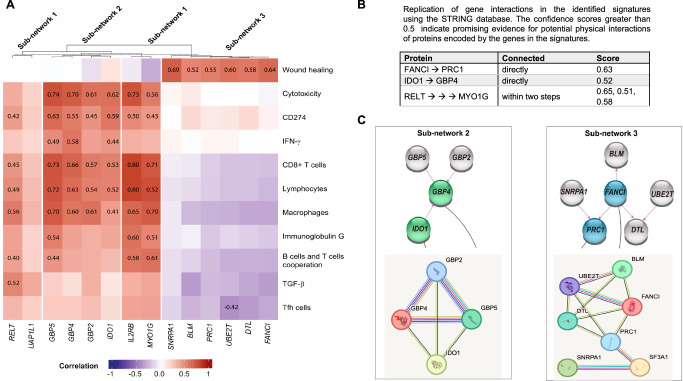
Functional relationships. **A** Immune related biological function of genes in the sub-networks 1-3. The heatmap shows the relationship between immune signatures and genes in the sub-networks with impact on OS. The correlations above ± 0.4 are presented on the heatmap. **B** Replication of gene interactions in the identified signatures using the STRING database. The confidence scores greater than 0.5 indicate promising evidence for potential physical interactions of proteins encoded by the genes in the signatures. **C** Representing the protein-protein interactions of the genes in subnetworks 2 and 3, where genes in their corresponding signatures show direct protein interactions according to STRING^15^. The upper subnetworks are identified in this data-driven study, while the lower subnetworks are based on STRING. The colors of the edges in STRING represent different sources of evidence for the interactions.

The biological functions of the gene signatures were assessed by clustering genes in the corresponding sub-networks (Fig. 4A) based on 10 immune signatures known as key inputs to a description of the immune subtypes of cancer. These immune signatures including macrophages^34^, lymphocytes^35^, TGF-β^36^, IFN-γ^37^, wound healing^37^, and CD8+T cell^38^ measure a final common pathway of antitumor immune activity (cytotoxicity^38^), characteriz the immune microenvironment (T follicular helper, Tfh, cells^39^), and mediate the response to checkpoint inhibitors (B cells and T cells cooperation^40^).

The median value of the genes (Supplementary Data 3 and 4) within an immune signature was assigned to each patient except for the cytotoxicity signature where the geometric mean was used, according to Rooney et al.^41^. In addition, we included immunoglobulin G^42^ and single gene CD274^43^ as prognosis biomarkers. Since in this study, gene expression levels are adjusted for enriched immune cell types, we first investigated the relationship of the immune signatures and the enriched immune cell types (Fig. S6). We then clustered the genes in the sub-networks represented as a heat map plot in Fig. 4A.

As the heatmap in this figure shows, Sub-network 1 is divided into two clusters: one includes *IL2RB* and *MYO1G* and the other includes *UAP1L1* and *RELT*, consistent with what we observed in the OS analysis where *MYO1G* prolonged OS and *RELT* shortened OS. Both genes (*MYO1G* and *RELT)* in the corresponding signature, showed stronger correlation with macrophages.

All the gene in sub-network 2 are clustered together and represented strong correlation with cytotoxicity, CD8+T cells, lymphocytes, and macrophages. The two genes (*IDO1* and *GBP4*) in the corresponding signature showed stronger correlation with cytotoxicity (Fig. 4A).

On the other hand, sub-network 3 is only related to wound healing and all the genes in this pathway showed correlations with the wound healing (Fig. 4A).

### Replications

In addition to using 103 samples for replication of edges in the network, we used three external replication cohorts. We replicated the edges in the network and investigated the functional relationships among genes within identified signatures using the STRING database, a biological database of protein–protein interactions. The database integrates and compiles information on known and predicted protein-protein interactions from various sources, including experimental data, computational predictions, and literature mining^15^. Figure 4B represents confidence scores for protein interactions corresponding to the genes in the identified signatures based on data-driven approch in this study. Scores above 0.5 are indicative of promising evidence for potential physical interactions. Figure 4C represents the protein protein interactions of the genes in subnetwork 2 and 3, where genes in their coresponding signatures shows direct protein interactions according to STRING. The colors of the edges in STRING represent different sources of evidence for the interactions.

We used the GSE146889 data from the Gene Expression Omnibus database as an external cohort, which includes 24 paired samples from normal and tumor tissue of colorectal cancer patients. We performed differential expression analysis and observed significant upregulation of *RET*, *FANCI*, and *PRC1* (FC: 1.44, *p* value: 0.04; FC: 3.95, *p* value: < 0.0001; FC: 4.27, *p* value: < 0.0001).

We also replicated the gene-OS relationships using data from the COAD project of The Cancer Genome Atlas (TCGA), consisting of 27 samples treated with bevacizumab and ten with cetuximab. Thus, we performed the replication analysis for the findings related to the bevacizumab arm and not for cetuximab due to the limited number of samples. We used the Wilcoxon rank-sum test to validate the findings for the follow-up time of 27 patients treated with bevacizumab who exhibited elevated expression levels of both *GBP4* and *IDO1* or the absence of high expression levels in both genes. The expression levels were dichotomized based on the third quartile, similar to the Kaplan-Meier analysis. We observed a significant difference between these two groups (*p* value = 0.038), confirming the association of both *GBP4* and *IDO1* in sub-network 2 with the overall survival of patients treated with bevacizumab.

## Discussion

Predictive gene expression signatures may help to improve treatment assignments and provide guidance for a personalized treatment. In line with this goal, this study identified promising novel treatment-specific gene signatures for characterizing OS of patients under cetuximab or bevacizumab treatment. By dichotomizing the signatures to “high” vs. “medium-to-low” expression levels, we stratify patients according to their favorable response or resistance to these treatments. We replicated the results using different external databases at both gene expression and proteomic levels to increase the reproducibility of the findings and the likelihood of replication in future studies. Successful replication of the results in both internal and external cohorts was greatly influenced by considering the interconnectivity among genes using network analysis.

One of the identified gene signatures includes *IDO1* and *GBP4* that are typically expressed at low-to-medium basal levels in the absence of acute activating signals^44^. *IDO1*, with an important role in regulating the innate and adaptive immune response, is overexpressed in many types of cancers, including CRC^45^. Currently, an increasing number of studies have demonstrated that *IDO1* is associated with immune escape by suppressing T cell activity and enhancing regulatory T cells in different tumor types. However, this study revealed that an elevated level of *IDO1* transcription is associated with longer OS in patients treated with bevacizumab. This disparity has been reported in the study of gynecologic and breast cancers^46^, which might be due to the activation of tissue-specific gene regulatory pathways in tumor cells. Notably, this study suggests that the majority of patients with the CMS3 subtype exhibit medium to low expression levels of genes in this signature and are more likely to resist bevacizumab treatment with a median OS of 14.4 months (95% CI: 10.9–17.8).

The other signature includes *MYO1G* that constitutes the minor histocompatibility antigen HA-2 that binds to MHC class I molecules, makes the antigens recognizable by CD8+T cells in tumor cells, and allows the destruction of harmful tumor cells^47,48^. Interestingly, the OS analysis showed positive effects of high expression of *MYO1G* on OS in the cetuximab arm. Its upstream gene in the identified sub-network, *RELT*, is frequently overexpressed in colorectal cancer cell lines and primary colorectal carcinomas^49^, consistent with the negative effect of *RELT* on OS shown in this study. *RELT* activates NF-κB pathway and deregulates β-catenin activity in the majority of sporadic forms of colorectal cancer cell lines^49^.

The protein regulator of cytokinesis 1 (PRC1) in the signature corresponding to sub-network 3, reduced OS of patients treated with cetuximab, anti-EGFR therapy. *PRC1* plays an important role in the pathogenesis of various cancers, including colon cancer^50^. *PRC1* and all the genes in its sub-network showed relationships with wound healing signatures, most likely due to their common function in DNA damage and repair. DNA damage sensing and repair dysregulation causes genome instability and is a hallmark of many cancers^51^. Moreover, this study suggests that the majority of patients with the CMS2 subtype exhibit medium to low levels of both *FANCI* and *PRC1* and derive greater benefit from cetuximab treatment with a median OS of 39.2 months (95% CI: 34.9–45.6).

Future research that explores the interplay between vascular factors and gene expression may provide further insights into the mechanisms underlying response to bevacizumab. This is particularly relevant because bevacizumab primarily targets angiogenesis by inhibiting vascular endothelial growth factor (VEGF), resulting in significant vascular changes and adverse events^52^.

Overall, these findings underscore the potential for more targeted and effective therapeutic strategies in managing colorectal cancer. The signatures stratified patients who are more likely to respond positively to cetuximab or bevacizumab, as well as those who may exhibit resistance to these therapies. Further studies are needed to translate these findings into clinical practice. However, the use of data-driven networks has increased the likelihood of translation, as demonstrated by successful replications.

## Supplementary information

Supplementary methods and figures

Description of Additional Supplementary Files

Supplementary Data 1

Supplementary Data 2

Supplementary Data 3

Supplementary Data 4

Reporting Summary

## Data Availability

The gene expression data used in this study are publicly available in Gene Expression Omnibus at GSE196576. These data serve as the source for Figs. 2, 3, 4A. Additionally, Supplementary Data 2 and 3 were used for Fig. 4. The source data for Figure 5 is available at https://github.com/AkramYazdani/Optimal_alpha_for_Constructing_Causal_Network.

## Code availability

Software and the code for analysis associated with the current submission are available at Reporting Summary and https://github.com/AkramYazdani/Optimal_alpha_for_Constructing_Causal_Network.
