# Supplementary material for: Gene signatures derived from transcriptomic-causal networks stratify colorectal cancer patients for effective targeted therapy
Source: Commun Med (Lond). 2025 Jan 8;5:9. doi: 10.1038/s43856-024-00728-z (PMC11711454; doi:10.1038/s43856-024-00728-z)
Supplement: Supplementary file 1 — Supplementary methods and figures [file 43856_2024_728_MOESM1_ESM.pdf]

Gene signatures derived from transcriptomic-causal networks stratify colorectal cancer patients for effective targeted therapy

Akram Yazdani[1,2*], Heinz-Josef Lenz[3], Gianluigi Pillonetto[4], Raul Mendez-Giraldez[5], Azam Yazdani[6], Hanna Sanof[7,8], Reza Hadi[9], Esmat Samiei[10], Alan P Venook[11], Mark J Ratain[12], Naim Rashid[13], Benjamin G Vincent[14], Xueping Qu[15], Yujia Wen[16], Michael Kosorok[13], William F Symmans[17], John Paul Y.C. Shen[18], Michael S Lee[18], Scott Kopetz[18,19], Andrew B Nixon[20], Monica M Bertagnolli[21], Charles M Perou[8,22], Federico Innocenti[1]

Supplementary Methods

We first accounted for hidden factors such as batch effects and technical artifacts or cell type abundance using PEER factors. We selected optimal number of PEER factors based on maximal number of cis-eQTLs identified through eQTL analysis [2]. We performed the cis-eQTL analysis for different number of PEER factors ({1, …, 5, 10, 14, …, 20}) and selected the top eQTLs (permutated p-value < 0.05) for each model. As presented in **Figure S8**, model with 1 PEER factor have the maximum number of eQTLs.

Since model with one PEER factor was selecting the most, we investigated the overlapped of the set of top eQTL of each pair of models represented in **Figure S9** where each curve represents a comparison of a targeted model i across all other models represented by l_i in X-axis. $l_i = M_{p_j}$ if $j < i$ otherwise $l_i = M_{p_{j+1}}$ where p_j is jth number of PEER factor {1, …, 5, 10, 14, …, 20} correspond to jth model. **Figure S9** shows decline of the overlap between each pair is associated with the difference between the number of PEER factors. These results suggested that PEER factors do not capture variation in the data in this study which is also observed in variance partition analysis for 4 PEER factors as an example (**Figure S10**).

Supplementary Figures

Figure S1. Consort chart for germline genotype data

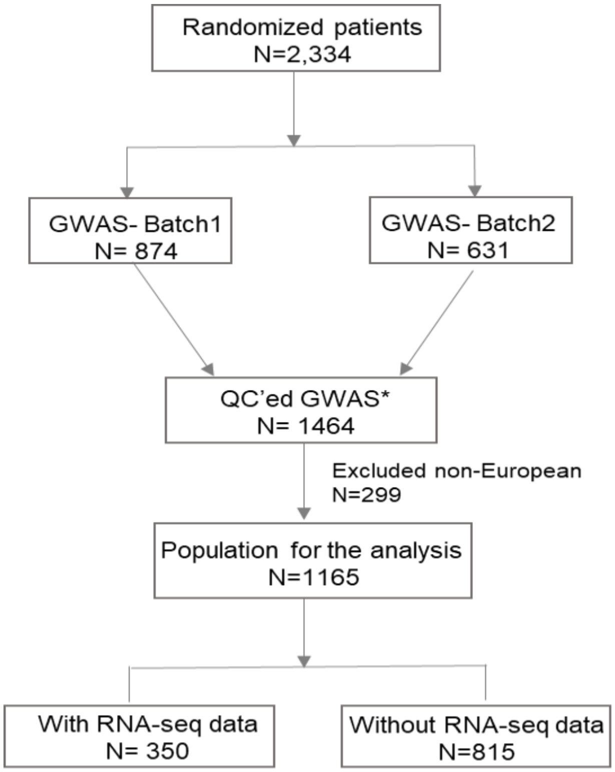

Figure S2. Consort chart for RNA-seq data

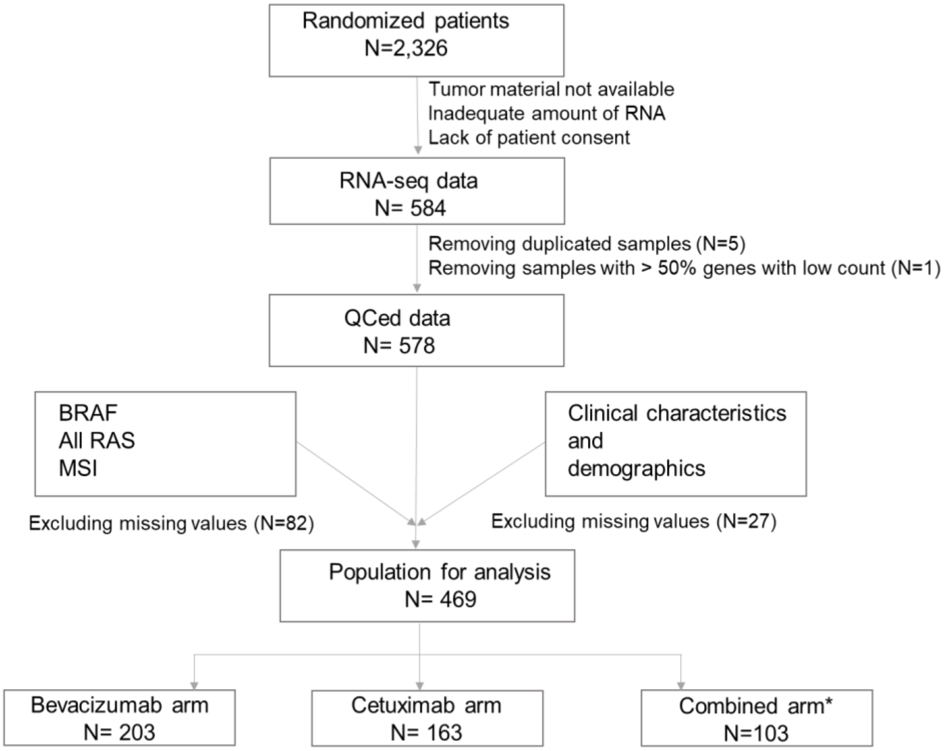

*Combined arm: Bevacizumab + Cetuximab + chemotherapy

Figure S3. Quality of the gene expression across samples using boxplot

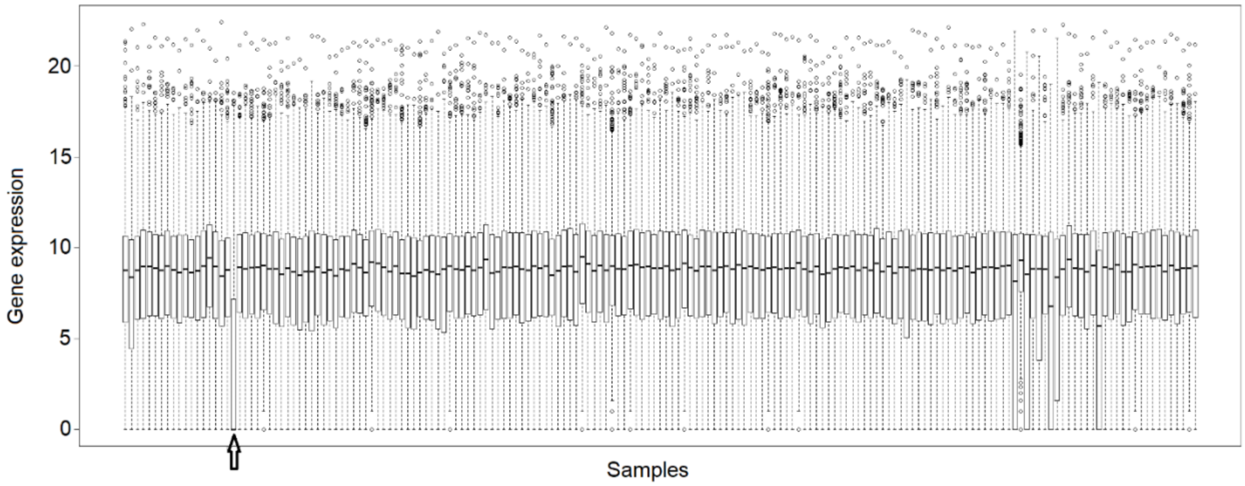

Figure S4. Principal component analysis to assess presents of batch effect on RNA-seq

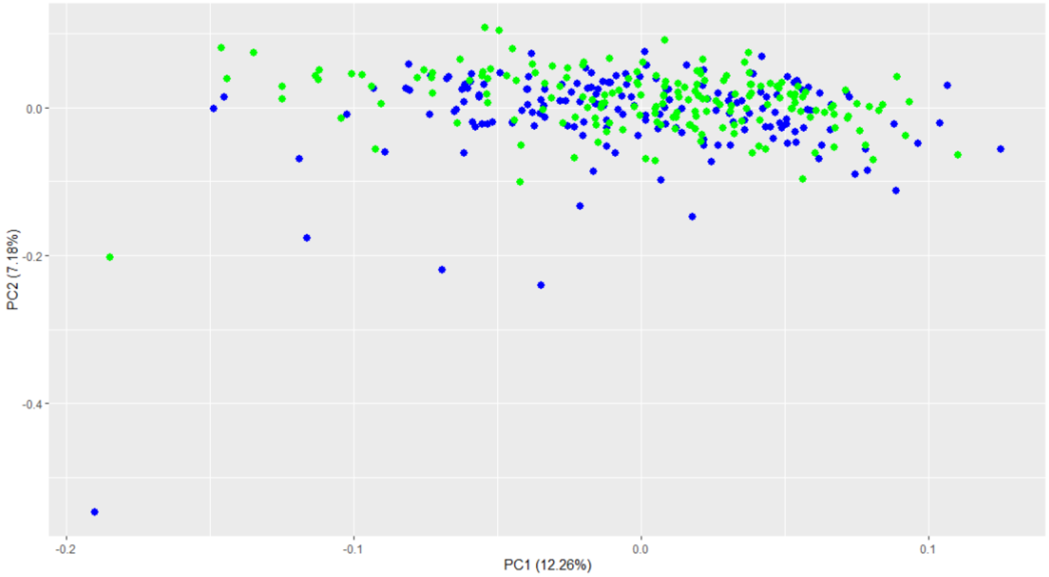

Figure S5. Gender verification using k means clustering of samples based on expression of genes in chromosome Y.

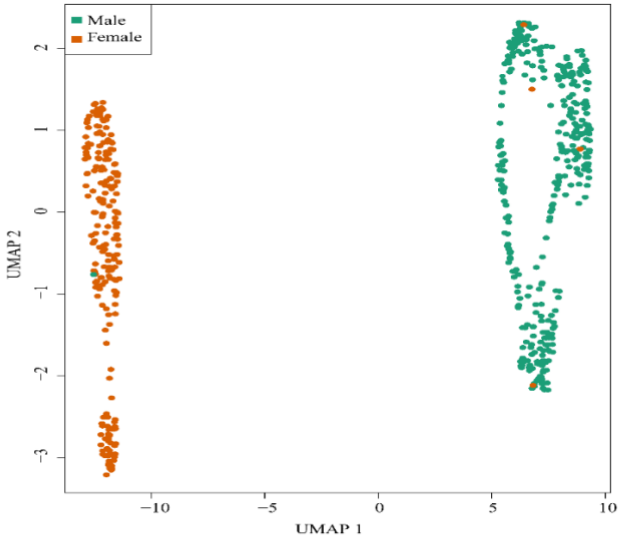

Figure S6. The relationship between immune features including estimated immune cell type abundant score and immune signatures.

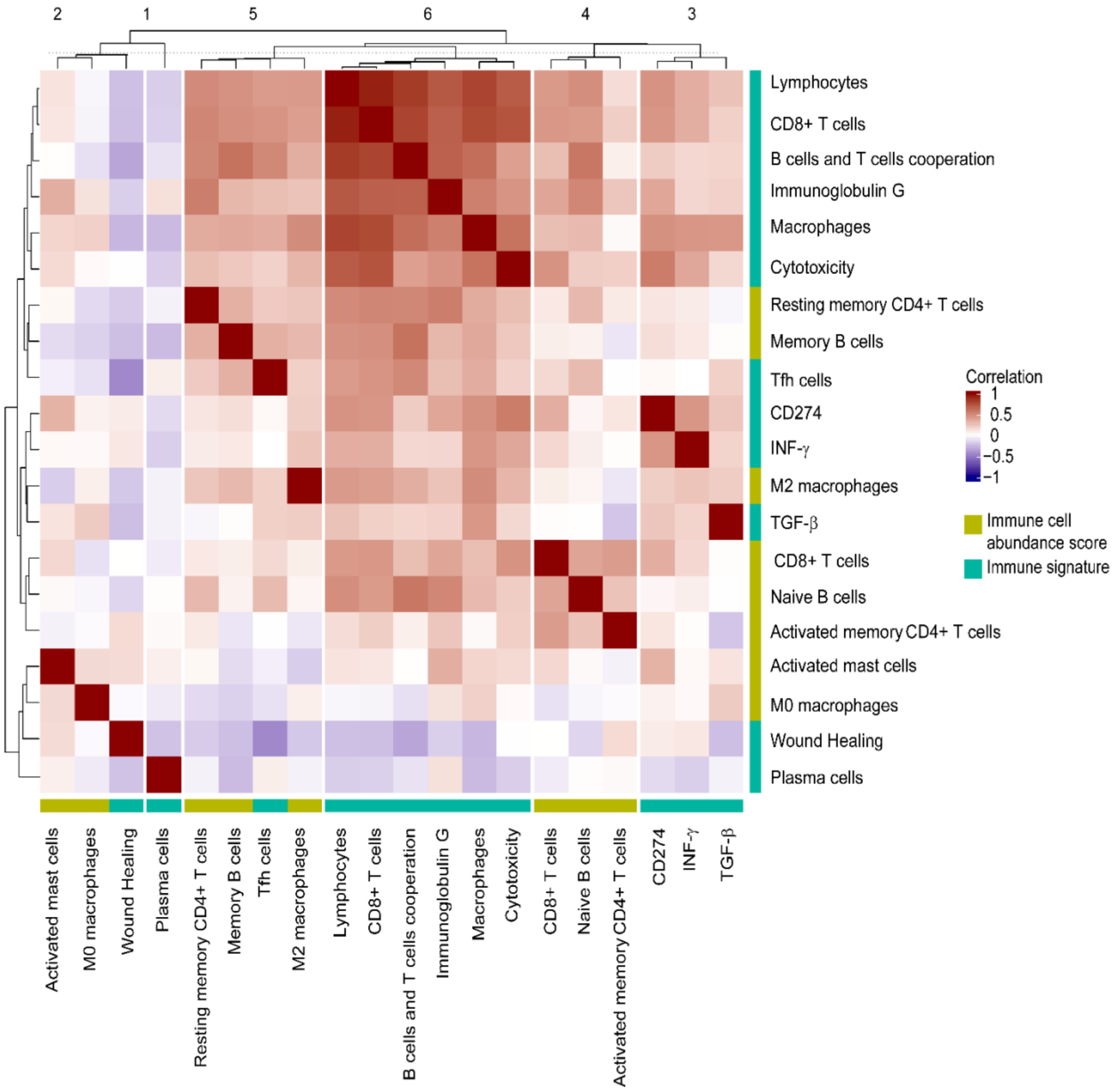

Figure S7. Estimation of overall survival for each treatment using Kaplan-Meier plot

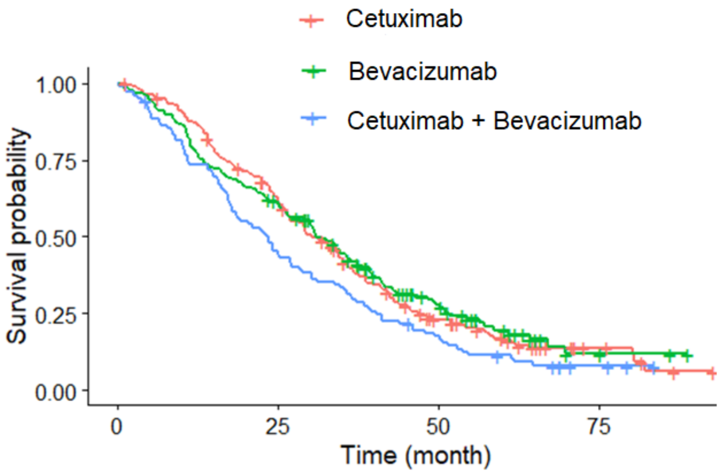

Figure S8. Bar plot for the number of significant eQTLs in the models with different numbers of PEER factors.

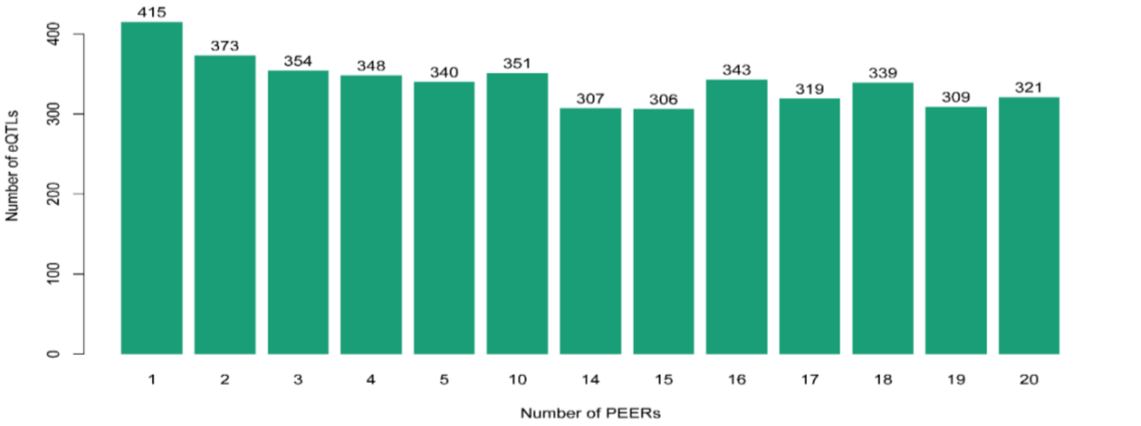

Figure S9. Comparing the number of overlapped eQTL identified with each pair of models. The peaks are related to the models with the lower number of differences in the PEER factors.

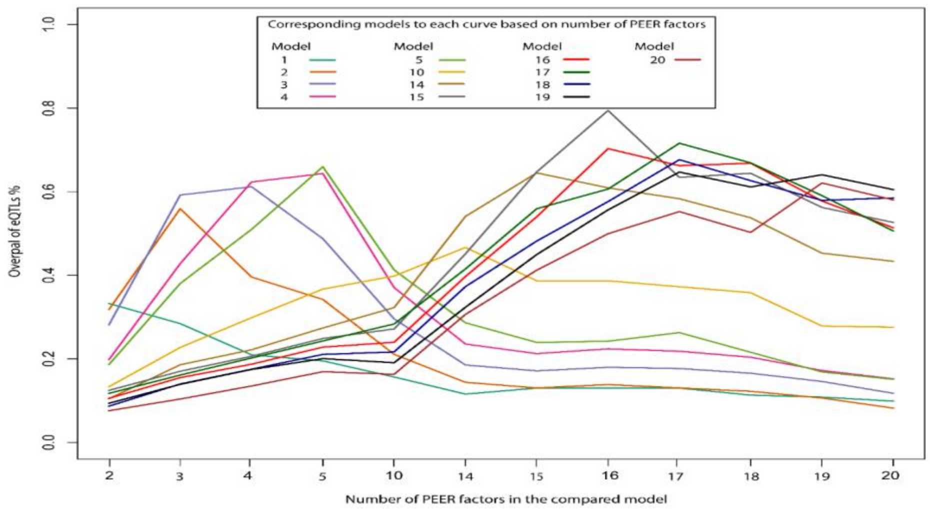

Figure S10. Variation in the RNA-seq data explained by 4 PEER factors.

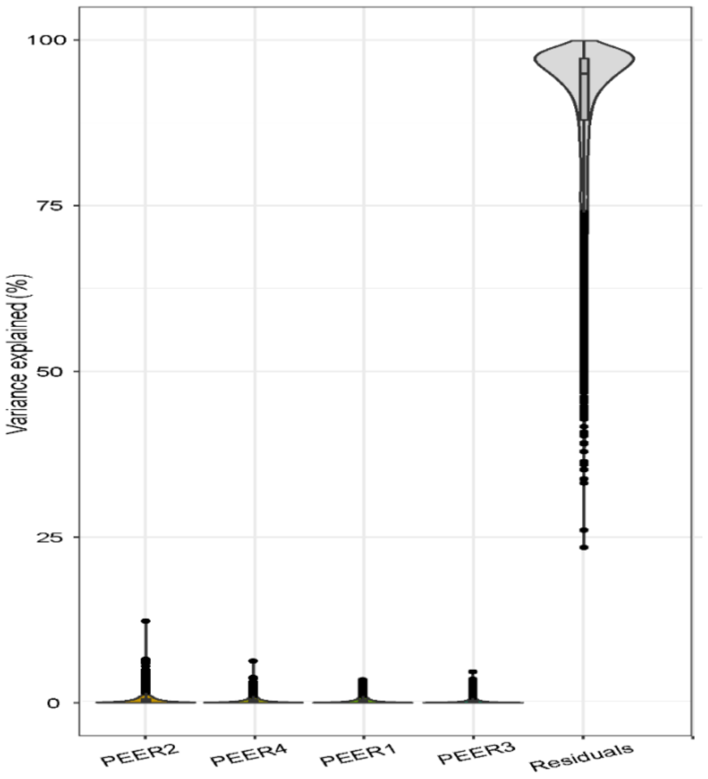

Figure S11. The correlation of first principal components of genotyped data with other covariates

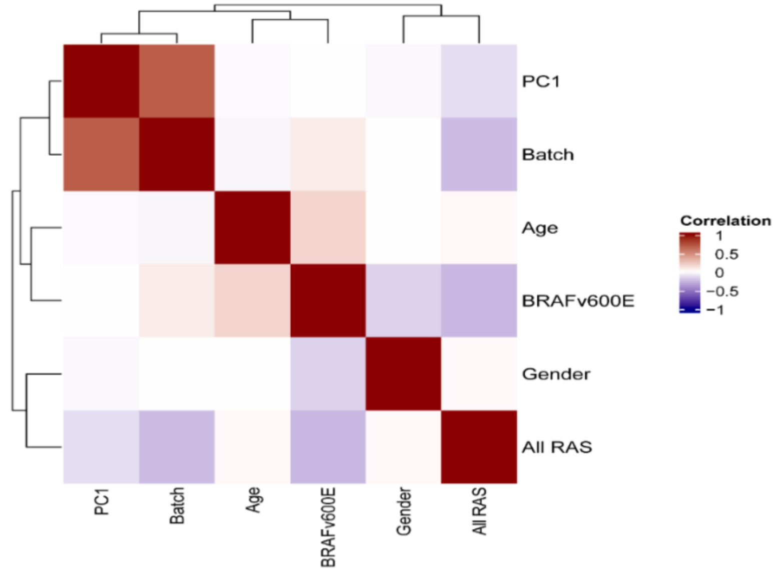

Figure S12. Piecewise regression for assessing stability of the networks based on the Hamming distance metric. Y-axis represents *p*-values from model (1) for coefficients β_3 (Methods). X-axis represents -log10 of α_i.

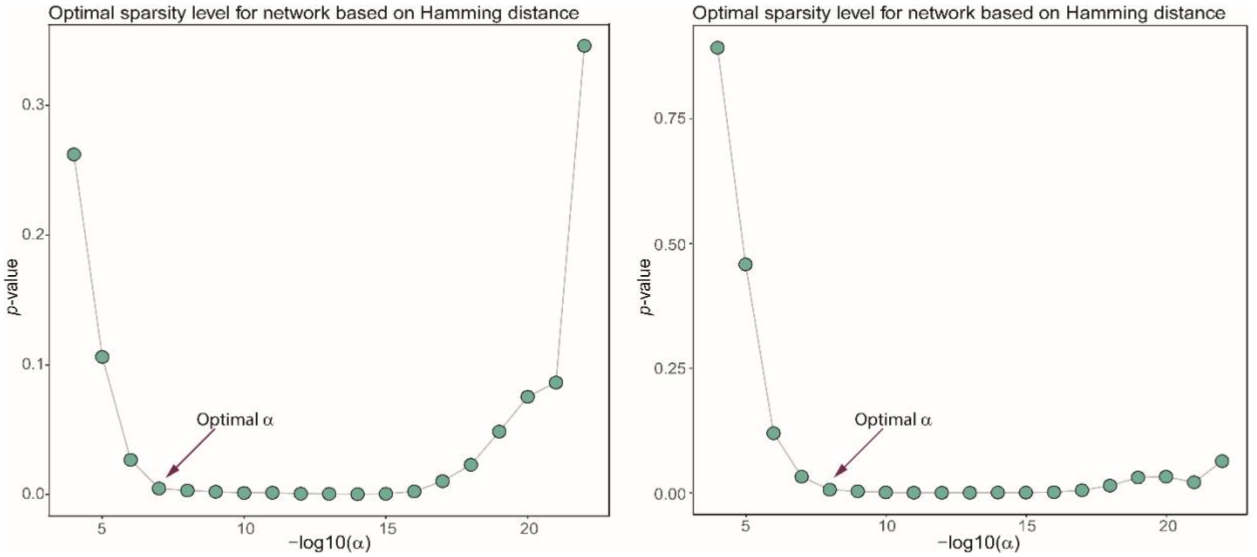

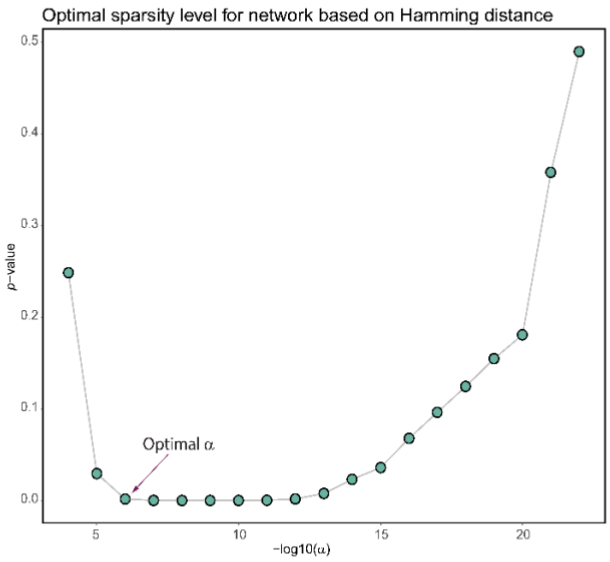

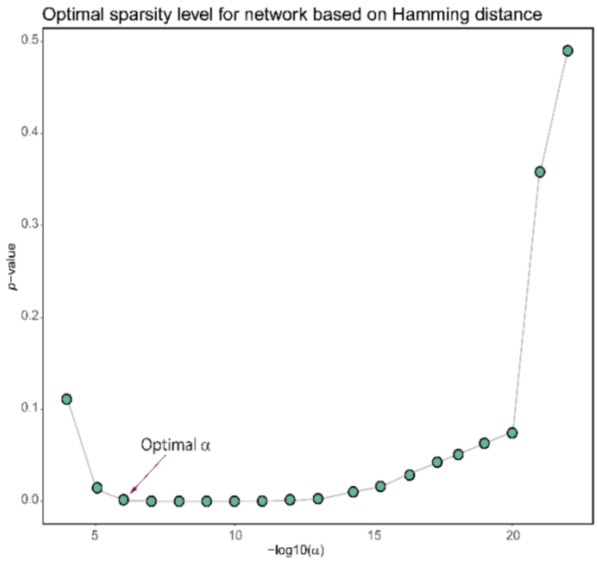

Figure S13. The illustration of a V-structure [3,4] that refers to a situation in which two genes are regulating a downstream gene as illustrated in Figure below. Assume three random variables, here three genes, $g_i, i = 1,2,3$ represented as nodes. The v-structure is identified when $g_1$is independent from g_2 ($g_1 \perp g_2$) but is not independent from g_2 given g_3 ($g_1 \not\perp g_2 |\ g_3$) which makes the probability distributions distinguishable and identifies a unique directionality from data, the arrows from g_1 and g_2 point to g_3.

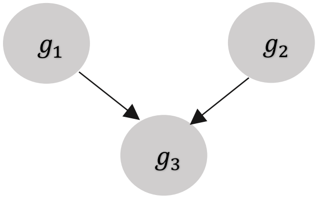

Figure S14. Histogram of the distance of each *cis*-eQTL from TSS of the gene

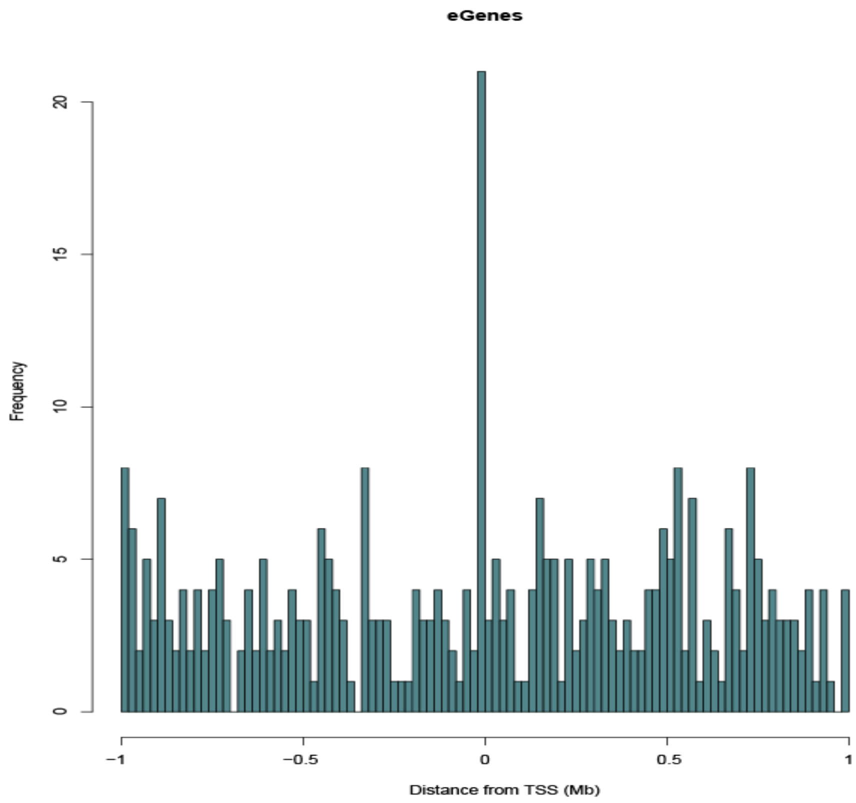

Figure S15. *P*-values of *cis*-eQTL vs the distance from TSS of the gene

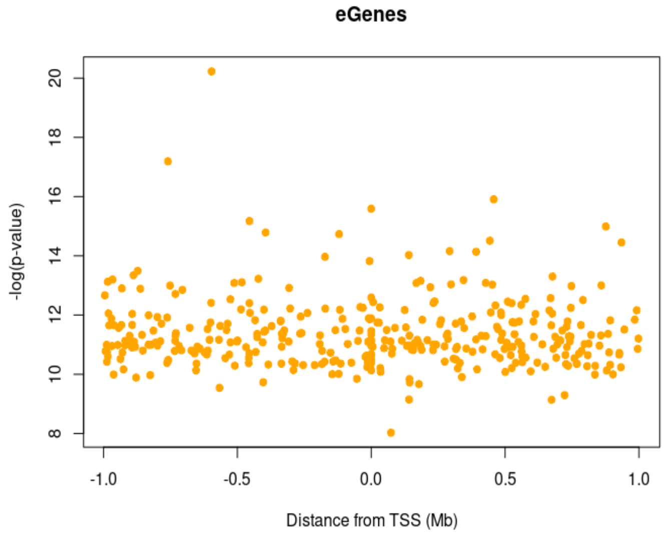

Figure S16. Enrichment analysis for *cis*-eQTL annotation. **A**. *Z*-score of the enrichment analysis for genomic location revealed a significant depletion of exons. **B**. *Z*-score of the overlap of *cis*-eQTLs with Roadmap enhancers for colon and rectum tissue; an active enhancer refers to the regulatory region of DNA that interacts with the promoter DNA region; a genic enhancer refers to regulatory regions in a gene; a bivalent enhancer refers to segments of DNA that have both repressing and activating epigenetic regulators in the same region. This analysis showed a high enrichment of cis-eQTLs in bivalent chromatin states associated with enhancer sequences based on the Roadmap Epigenomics Consortium enhancer databases [1].

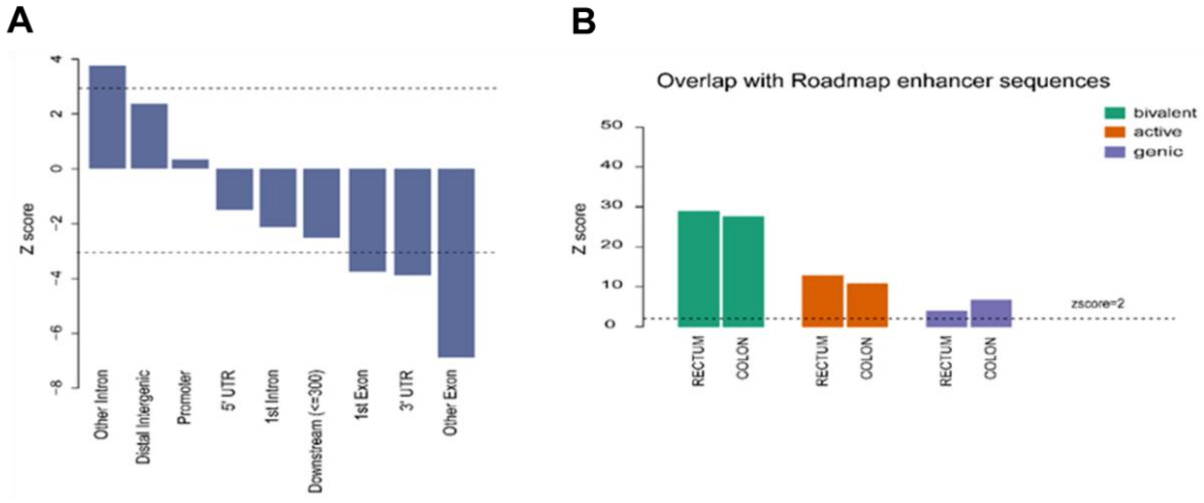

Figure S17. The Venn diagrams represent the number of samples with either RNA-seq or germline genotype data in each arm of the study.

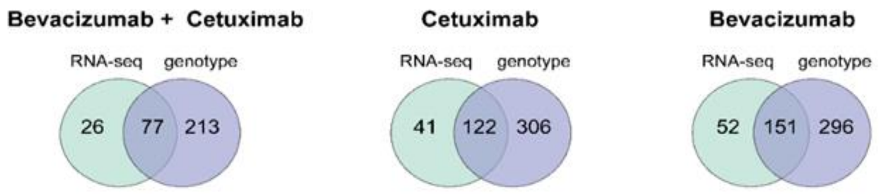

Figure S18. Prediction of the gene expression in the sub-network 1 given the relationship between the genes using data from the combined arm of therapy (cetuximab + bevacizumab).

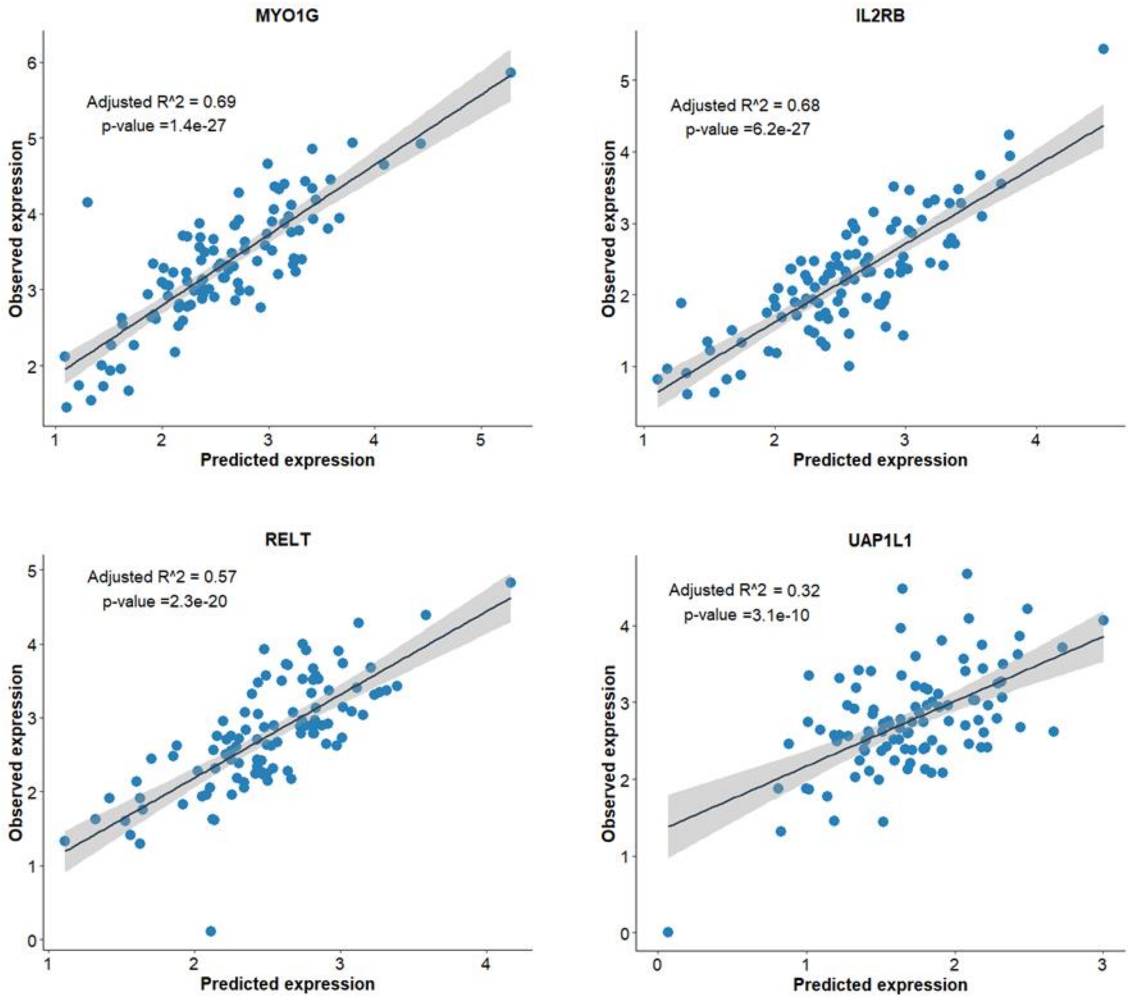

Figure S19. Prediction of the gene expression in the sub-network 2 given the relationship between the genes using data from the combined arm of therapy (cetuximab + bevacizumab).

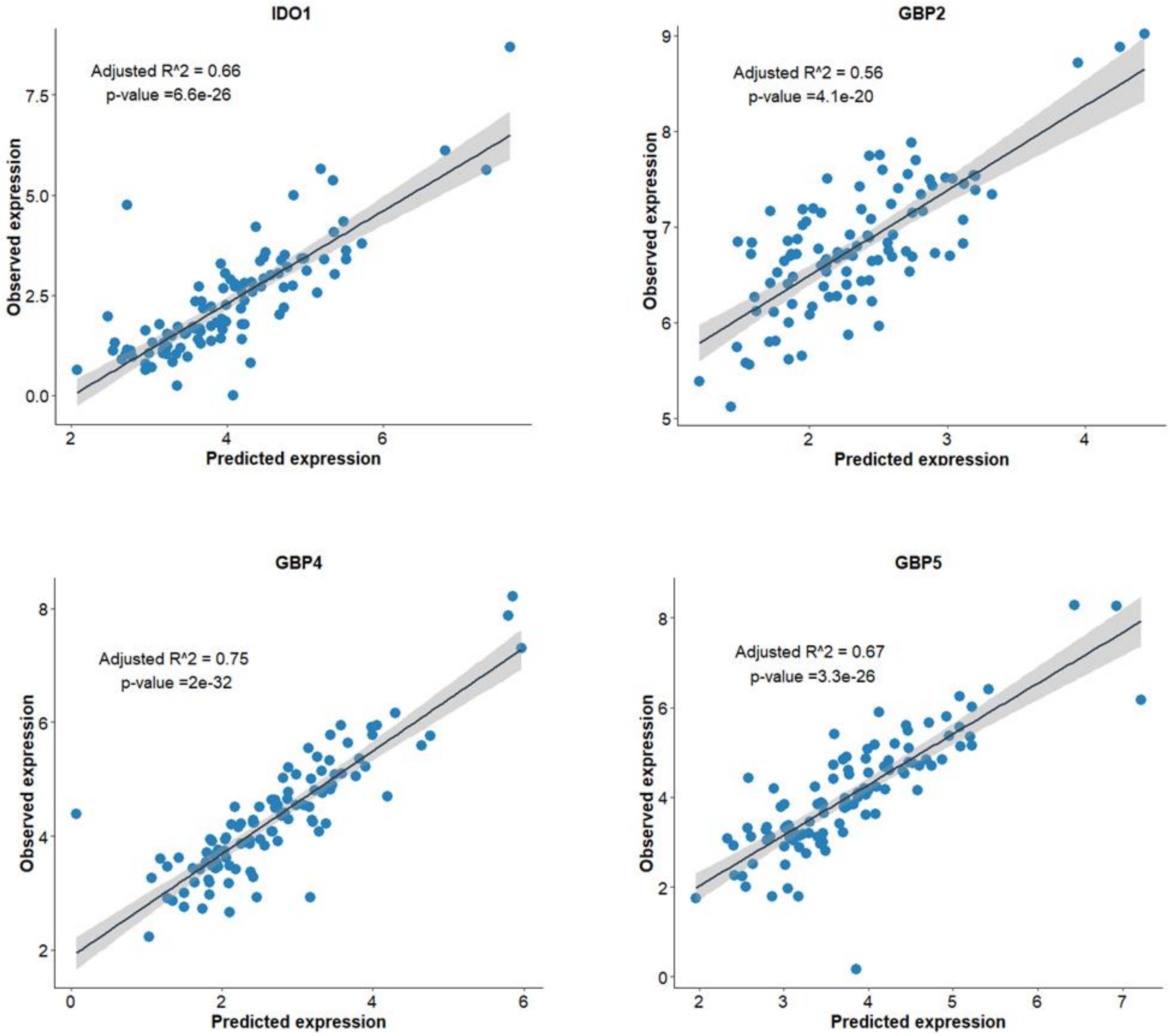

Figure S20. Prediction of the gene expression in the sub-network 3 given the relationship between the genes using data from the combined arm of therapy (cetuximab + bevacizumab).

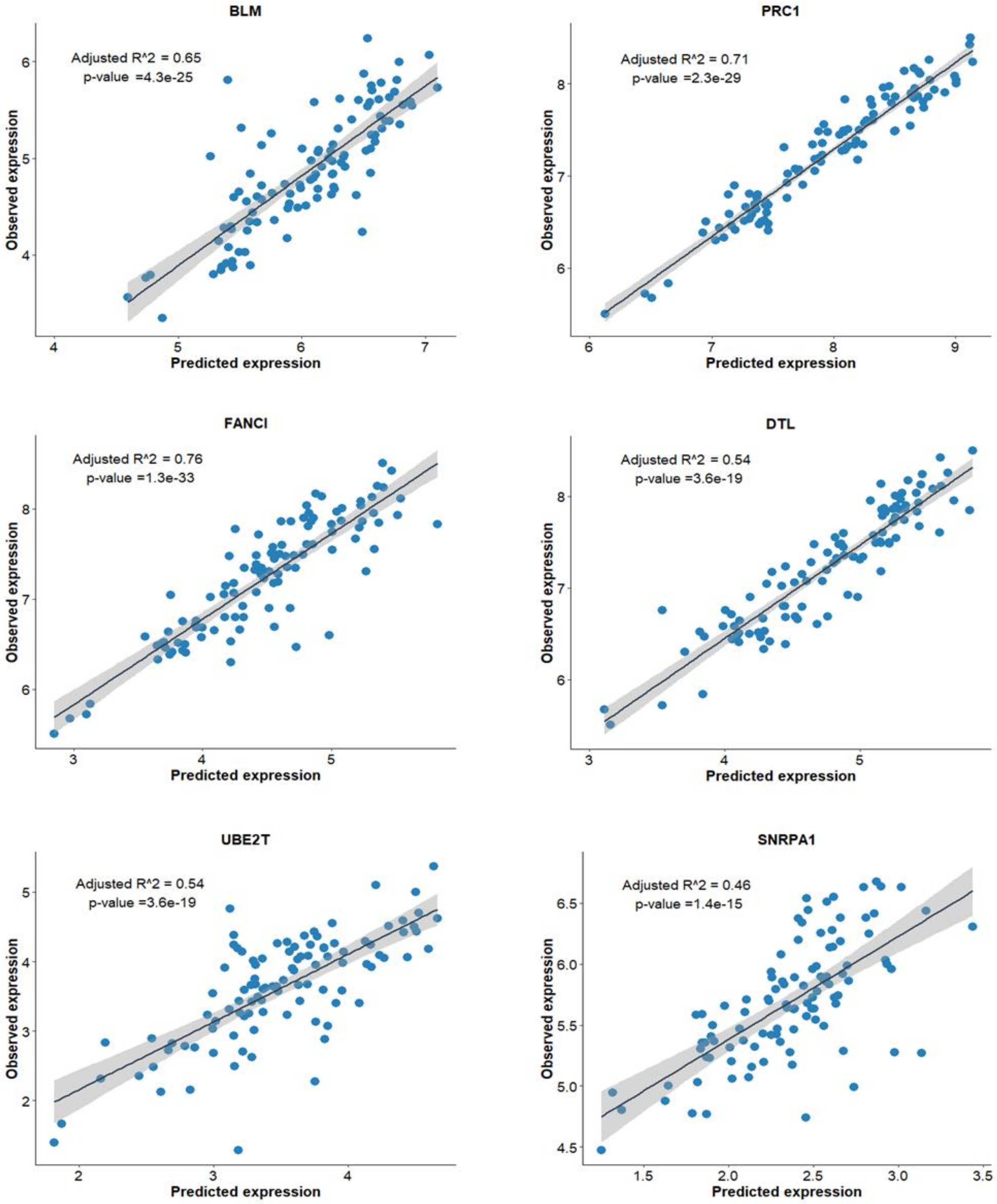

Figure S21. Kaplan-Meier plots for signatures 1 and 3, "high" indicates signature with both genes at high expression level, "medium-to-low" indicates both genes are at medium-to-low expression levels, "high or medium-to-low" indicates genes are either at high or medium-to-low expression levels. The high and medium-to-low for each gene defined based on the third quartile value of expression level across all the samples in the cohort.

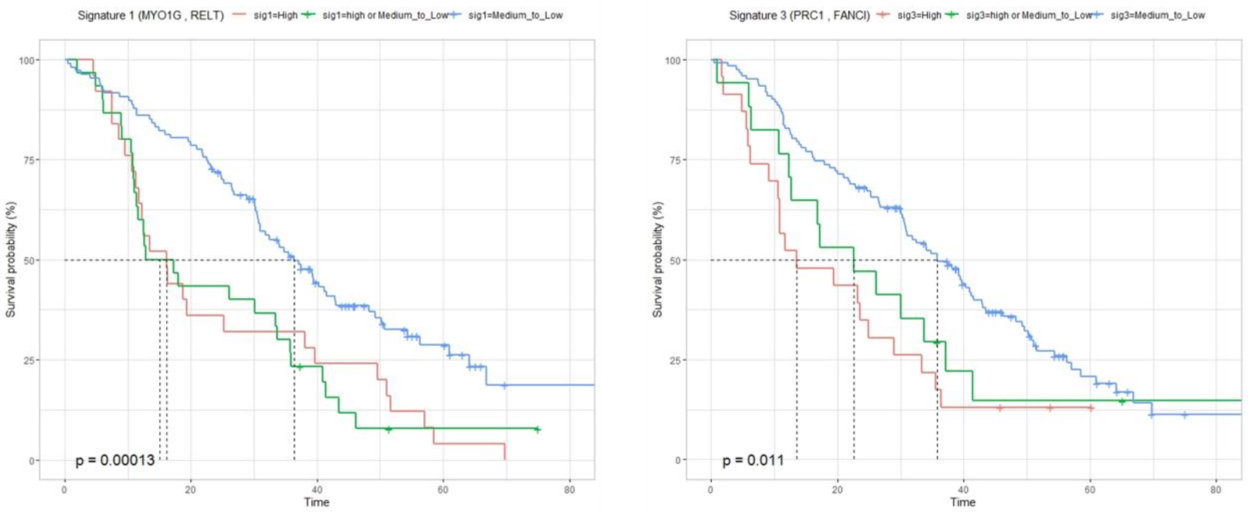

Supplementary References

[1] Roadmap Epigenomics Consortium, Kundaje A, Meuleman W, et al. Integrative analysis of 111 reference human epigenomes. Nature. 2015;518.

[2] Stegle O, Parts L, Durbin R, et al. A bayesian framework to account for complex non-genetic factors in gene expression levels greatly increases power in eQTL studies. PLoS Comput Biol. 2010;6.

[3] Pearl J. Causality. New York: Cambridge. 2000;

[4] Verma T, Pearl J. An Algorithm for Deciding if a Set of Observed Independencies Has a Causal Explanation. Uncertainty in Artificial Intelligence. 1992.
