# Supplementary material for: Gene signatures derived from transcriptomic-causal networks stratify colorectal cancer patients for effective targeted therapy
Source: Commun Med (Lond). 2025 Jan 8;5:9. doi: 10.1038/s43856-024-00728-z (PMC11711454; doi:10.1038/s43856-024-00728-z)
Supplement: Supplementary file 2 — Description of Additional Supplementary Files [file 43856_2024_728_MOESM2_ESM.pdf]

Description of Additional Supplementary Files

File name: Supplementary Data 1

File description: The comparisons between the population with the RNA-seq and the population without it.

File name: Supplementary Data 2

File description: The permutation test for the relationship of cis-eQTL-gene pairs assessed using 350 samples with both RNA-seq and genotype data.

File name: Supplementary Data 3

File description: The median value of the genes within an immune signature.

File name: Supplementary Data 4

File description: The median value of the genes within an immune signature.
